# The Nutritional, Glutathione and Oxidant Status of Elderly Subjects Admitted to a University Hospital

**DOI:** 10.4103/1319-3767.74474

**Published:** 2011

**Authors:** Adel A. Alhamdan, Abdulaziz A. Alsaif

**Affiliations:** Clinical Nutrition Program, Community Health Sciences Department, College of Applied Medical Sciences, King Saud University, Saudi Arabia; 1 Department of Surgery, College of Medicine, King Saud University, Saudi Arabia

**Keywords:** Old subjects, nutritional status, glutathione, thiobarbituric acid-reactive substances

## Abstract

**Background/Aim:**

Malnutrition in elderly patients is common in hospitals, and many of the age associated chronic diseases have a common factor, which is oxidative stress. The aim of the study was to evaluate the nutritional status, glutathione, and oxidant status of elderly patients.

**Patients and Methods:**

The mini-nutritional assessment (MNA) was used to determine the nutritional status of elderly patients. Glutathione concentration in the whole blood, plasma albumin, and thiobarbituric acid-reactive substances (TBARS) levels was measured spectrophotometrically by the enzymatic recycling method. In addition, length of hospital stay was estimated. All measurements were taken within 48 h after admission.

**Results:**

The results showed that more than two-thirds of the elderly were classified as at risk of malnutrition and malnourished. About 45% and 53% elevation in the TBARS was found in at risk of being malnourished and in the malnourished groups, respectively, compared to the well-nourished group, but the elevation did not reach the significant level. No significant differences in the glutathione concentration and in the length of hospital stay were found among the three mini-nutritional assessment categories.

**Conclusions:**

The study indicated the necessity of performing the MNA test for elderly upon admission to hospitals, and more attention needs to be paid to this vulnerable group of people.

Nutritional status plays an important role in the quality of health of elderly people. Aging is a risk factor for protein energy malnutrition. Poor nutritional status is one of the major factors associated with mortality in older subjects.[1] A high prevalence of undernutrition has been reported in hospitalized and institutionalized elderly patients.[2] Malnutrition in elderly patients is common in hospitals, leading to prolonged hospital stay and imposing large costs on health services.[3] An elderly patient who is malnourished when admitted to hospital tends to have a longer hospital stay and have more complications than a well nourished-patient having the same disease.[4] Elderly are at an increased risk of undernutrition because of inadequate food intake, reduced desire to eat, decline in food digestion or absorption, compromise in nutritional metabolic pathways, poor dental hygiene, or chronic diseases that are commonly found in elderly.[5,6] Furthermore, undernutrition impairs immunity and reduces the antioxidant status of the body.[7]

Although the majority of persons aged 70 years and older are reported to be in good to excellent health,[8,9] it is estimated that 85% of noninstitutionalized older persons have one or more chronic conditions that could improve with proper nutrition[10,11] and that up to half have clinically identifiable problems that require nutrition intervention.[9,10,12]

Reactive oxygen species, which is a general scientific term usually used to refer to free and non-radical molecules that are able to oxidize other biomolecules, or are easily converted into radicals, have been shown to be a common factor in many of the age-associated chronic diseases e.g. rheumatoid arthritis, heart disease, and Alzheimer disease.[13–15] Thus, improving the nutritional status of elderly subjects and maintaining adequate amounts of antioxidant defenses, such as glutathione (GSH), which is one of the major antioxidant compounds within the cell, could play a major role in improving the overall health of elderly subjects by protecting the body from these potentially toxic molecules.

Glutathione has several major physiological functions: (1) protecting cells against destructive effects of reactive oxygen intermediates and free radicals; (2) detoxifying external substances such as drugs and environmental pollutants; (3) maintaining red cell membrane stability; and (4) enhancing immunological function through its effects on lymphocytes.[16,17] In a sample of clinically healthy groups, glutathione levels were significantly lower in old (60-79 years) when compared to mature (40-59 years) subjects. However, levels in the very old (80-99 years) were similar to those of the mature group. This curvilinear pattern suggests that this selected sample of very old healthy men and women may represent survivors with extreme longevity.[18] In another convenience sample of elderly white women, selected on the basis of good health, blood glutathione levels were found to be the highest ever observed.[19]

Two biochemical perturbations resulting from oxidative stress are lipid peroxidation and protein carbonyl formation. Lipid peroxidation, measured as thiobarbituric acid-reactive substances (TBARS), has been used as a biomarker in a number of studies.[20–22]

The MNA (Mini Nutritional Assessment)[23] has been an extensively used method to identify risk of malnutrition in the elderly and in those that may benefit from early intervention. The MNA is a simple, low cost, and non-invasive method that can be done at bedside.[23] Added MNA scores allow one to screen the elderly who have an adequate nutritional status, those who are at risk of malnutrition, and those who are malnourished. The MNA consists of anthropometric and global indicators, including information on eating patterns and self-perception of health, such as reduced food intake; weight loss of >3 kg body weight; mobility, bed- or chair-bound; psychological stress; neuropsychological problems; body mass index; inability to live independently; taking >3 prescription drugs; having pressure sores or skin ulcers; number of full meals eaten per day; consumption of high-protein foods; consumption of fruits and vegetables; amount of liquids consumed per day; inability to feed self; difficulty in self-feeding; self-view of nutritional status; self-view of health status; mid-arm circumference <21 cm; and calf circumference <31 cm.[23]

In this study, the MNA was used to determine the nutritional status of elderly subjects upon admission to King Khalid University Hospital (KKUH), Riyadh, Kingdom of Saudi Arabia. GSH concentration in the whole blood, thiobarbituric acid-reactive substances (TBARS), as a marker of oxidative stress, and plasma albumin levels were measured. In addition, length of stay (LOS) in hospital was estimated.

## PATIENTS AND METHODS

Elderly patients (≥ 60 years old) admitted to medical wards between May and November, 2004 (n=100) in KKUH were the target subjects of the study. Eighty-five subjects were included in the study, after excluding 15 subjects because of various reasons (e.g. smoking, unable to complete the questionnaire, refused to give blood after writing the consent form, discharge before 48 h of admission). The study was approved by the ethical committee of the hospital, and subjects gave informed consent of the study. The mean age of the elderly was 71.4 ± 9.40 years (range 60-100 years), and 55 (64.7%) were female.

The MNA was used to determine the nutritional status of elderly subjects.[24] The MNA is a well-validated tool, with high sensitivity, specificity and reliability, specifically designed for assessing the nutritional status of elderly subjects in outpatient clinics, nursing homes, and hospitals.[25,26] The MNA scale (0-30 points) consisting of 18 point-weighted questions, composed of anthropometric measurements, global assessment, dietary questionnaires, and subjective assessment. The answers can give a maximum of 30 points. Depending on the score of the test, elderly subjects involved in the study were classified in the following categories: well-nourished (MNA points 24-30), at risk of malnutrition (MNA points 17-23.5), and undernutrition (MNA < 17 points).

Measurements of weight (to the nearest 0.1 kg) and height (to the nearest 0.1 cm) were made using a portable scale and a portable stadiometer, respectively. Body mass index (BMI) was calculated by dividing the weight in kilograms by the square of the stature in meters (kg/m^2^). Calf circumference was taken with an insertion measuring tape, while the elderly is lying supine, and the left knee and ankle were bent to a 90° angle. The loop of the tape is moved up and down the calf to locate the largest diameter, and the measurement was recorded to the nearest 0.1 cm. The anthropometric-measurement section of the MNA was taken by one well-trained nurse, and the remaining sections were taken by two nurses.

LOS was determined from the day of admission to the hospital to the moment of discharge.

Total GSH in the whole blood was measured in freshly collected blood samples by the enzymatic recycling method.[27,28] The Kit used to analyze GSH was purchased from Cayman Chemical Company, USA.

The total amount of lipid peroxidation products, as an indication of oxidative stress, present in the plasma samples of elderly subjects, were measured as reaction products of malondialdehyde (a compound generated as a result of lipid oxidation) with thiobarbituric acid. These products are called TBARS. The formation of TBARS was measured at 532 nm using a standard spectrophotometer.[45] The Kit used to analyze TBARS was purchased from ZeptoMetrix Corporation, USA.

Biochemical measurements such as serum albumin and total cholesterol are well known markers for protein energy malnutrition (PEM).[29,30]

Albumin concentration, as a marker of chronic-protein malnutrition, was measured in plasma using albumin kit (Albumin Flex^®^ reagent cartridge) purchased from Dade Behring Limited, UK. The albumin method is an adaptation of the bromocresol purple dye-binding method.[31,32]

### Statistical analyses

Results were expressed as mean (M) ± standard deviation (SD), and in number and percentage. When the mean values were compared, one-way analysis of variance (one-way ANOVA) was used. Differences in mean values with *P*< 0.05 were considered significant. Where significant effects were found, differences between groups were examined using Tukey’s test for multiple comparisons with a significant level of *P*< 0.05. Logarithmic transformations were made to normalize LOS.

## RESULTS

Of the 85 elderly subjects enrolled in the study, more than 85% of the subjects were found either to be at risk of malnutrition or malnourished [Table 1]. Elderly people, who were classified as malnourished, according to the MNA score, had the lowest BMI and albumin [Table 2]. A significant difference in the BMI and albumin values was observed between those who were classified as at risk of being malnourished and those classified as well nourished. When the BMI was classified into underweight (< 18.5 kg/m^2^), normal weight (18.5-24.9 kg/m^2^), overweight (25-29.9 kg/m^2^), and obese (> 30 kg/m^2^), based on the World Health Organization classification,[42] it was found that 2.4%, 29.4%, 32.9%, and 35.3% of the subjects were underweight, normal weight, overweight, and obese, respectively.

**Table 1:** The mini-nutritional assessment score, number, and percentage of elderly subjects in the three MNA categories upon admission to medical wards

MNA categories	MNA score	n	%
Well-Nourished MNA ≥24 points	26.5 ±1.4	11	12.9
At risk of malnutrition MNA 17-23.5 points	20.1 ±2.1	43	50.6
Malnourished MNA <17 points	14.6 ±1.8	31	36.5

MNA: Mini-nutritional assessment, Results are presented as mean ± SD, and in number (n) and percentage (%)

**Table 2:** Body mass index, plasma concentrations of albumin and thiobarbituric acid reactive substances, and whole blood glutathione level of elderly subjects in the three mini-nutritional assessment categories, upon admission to medical wards

	Well-nourished MNA ≥ 24 points	At risk of malnutrition MNA 17-23.5 points	Malnourished MNA < 17 points	One-way ANOVA *P* value
BMI (kg/m^2^)	31.6 ± 6.1^a^	29.2 ± 6.6^a^	26.2 ± 6.0^b^	0.035
Albumin (g/l)	33.0 ± 5.7^a^	30.8 ± 5.1^a^	27.2 ± 4.2^b^	0.001
GSH (µmol/l)	958 ± 42^a^	1035 ± 295^a^	1013 ± 236^a^	0.712
TBARS (nmol/ml)	10.8 ± 6.9^a^	15.7 ± 9.7^a^	16.5 ± 8.2^a^	0.218

MNA: mini-nutritional assessment; BMI: body mass index; GSH: glutathione, TBARS: thiobarbituric acid reactive substances. Results are presented as mean ± standard deviation. Results were compared using one-way analysis of variance (one-way ANOVA), followed by Tukey’s multiple comparison. Means (row) having different letter superscripts following the number differ signifi cantly (*P*<0.05)

No significant difference in the GSH concentration was found among the three MNA categories [Table 2]. There was an increase in the TBARS values, in the at risk of being malnourished group and to a greater extent in the malnourished group compared to the well-nourished group, but the elevation did not reach a significant level [Table 2].

LOS was longer, but not statistically significant, in the at risk of being malnourished group (10.9 ± 9.1days) and in the malnourished group (11.0 ± 10.40 days) compared to the well-nourished group (7.7 ± 6.4 days), [median: 8 days for the well-nourished group vs. 7.5 and 6 days for the at risk of being malnourished and malnourished groups, respectively)].

When the subjects were classified according to their main diagnosis, no significant difference was found in the BMI and MNA score among the various diagnosed groups [Table 3]. The mean values of the MNA points, in all main diagnosed groups, were within the category of the “at risk of malnutrition.” Elderly subjects classified as malnourished or at risk of being malnourished presented more than 80% of total subjects on each main diagnosed group.

**Table 3:** The mini-nutritional assessment and body mass index in the elderly subjects admitted to medical wards according to their main diagnosis

Diagnosis group	n	MNA	MNA	MNA	BMI (kg/m^2^)	MNA score
		<17 n	17-23.5 n	≥24 n		
Pulmonary diseases	21	5	12	4	27.9 ±6.1^a^	20.23 ±4.84^a^
Cardiovascular diseases	38	14	19	5	29.5 ±6.3^a^	18.93 ±4.05^a^
Gastro-intestinal diseases	6	3	2	1	25.4 ±6.7^a^	17.50 ±4.40^a^
Others diseases*	20	10	9	1	27.3 ±4.8^a^	17.95 ±4.40^a^

BMI: body mass index; MNA: mini-nutritional assessment. Results are presented in number (n) to determine the number of subjects on each MNA classifi cation by their main diagnostic group, and in mean ± SD for the BMI and MNA score. The results for the BMI and MNA score were compared using one-way analysis of variance (one-way ANOVA), followed by Tukey’s multiple comparison. Means (column) having different letter superscripts following the number differ signifi cantly (*P*<0.05). *P*< 0.435 for the BMI and *P*< 0.311 for the MNA score.

* None of the other diseases group (e.g. cancer, liver, urinary, neuro-psychiatric, orthopedic diseases, and endocrine disorders) comprising more than five subjects

In the global assessment section of the MNA questionnaire, 75 subjects (82.3%), out of the 85 subjects enrolled in the study, recorded that they were taking more than three prescription drugs a day. In the dietary assessment section of the MNA, 60 subjects (70.6%) were found to eat three full meals a day, 23 subjects (27.0%) eating two full meals a day, and 2 subjects (2.4%) were found to eat only one full meal a day, before admission to hospital. Only 40 subjects (47%) stated that they were consuming at least one serving of dairy products/day, eating two or more servings of beans or eggs/week and consuming meat, fish, or poultry every day. Seventy-three subjects (85.9%) were found to consume two or more servings of fruits or vegetables/day. Fourteen subjects (16.5%) said that they required assistant while eating, 10 subjects (11.8%) said that they fed themselves but with some difficulties, and 61 subjects (71.7%) said that they fed themselves without any problem. Twenty-four subjects (28.2%) experienced a decline in the food intake over the past 3 months due to loss of appetite, digestive problems, or chewing or swallowing problems. Most of the elderly (89.4%) stated they were consuming fluid less than 5 cups a day.

## DISCUSSION

The study was performed to assess the nutritional status of elderly subjects admitted to medical wards at KKUH using the MNA and to evaluate antioxidant/oxidant status by measuring GSH and TBARS concentrations.

Assessment of nutritional status, as a part of screening protocols, is necessary to identify malnutrition, which is a potential cause or an aggravation of morbidity and mortality.[33] Using the MNA, the results showed that more than one-fourth of the subjects were malnourished, and nearly half of the subjects were at the zone of borderline of being malnourished, upon admission. The high percentage of elderly subjects classified as malnourished and at risk of being malnourished may indicate bad dietary habits. Rubenstein and his colleagues suggested that elderly subjects categorized as at risk of being malnourished or malnourished using the MNA test should receive additional medical and nutritional assessments and then plan interventions.[34]

It has been suggested that BMI ranges between 24 and 29 is more appropriate for the elderly population.[35] The mean value for the BMI in the study was within this range 28.2 ± 6.54. Body mass index is a method of defining undernutrition and has been extrapolated as a single assessment of nutritional status.[36]

Elderly people, who were classified as malnourished, according to the MNA score, showed the lowest albumin levels. Herrmann *et al*.[37] showed that in elderly hospitalized patients, low albumin levels were associated with longer hospital stays and increased the risk for 1-year readmission in the hospital.

Poor nutritional status on admission may increase the risk of longer hospitalization, as supported by other studies.[38,39]

The results showed that there was a 45% and 53% increase in the plasma TBARS levels in the at risk of being malnourished and the malnourished groups, respectively, compared to the well-nourished group, which may give an indication of oxidative stress, but it could be the small number of subjects enrolled in the study not allowing the elevation to reach the significant level. The results are in agreement with other previous works. In a study, the plasma concentration of TBARS increased significantly, 20–45%, in individuals of over 50 years of age, as compared with the 20–29 years group.[40]

In another study, the plasma TBARS were significantly increased in elderly in comparison with values reported in younger adults.[41]

The study is limited by the small study size. The length of stay for all patients showed a wide dispersal, thus, a better focus on extended stay patients in the future study. In addition, only GSH concentration, as an indication of antioxidant status was measured, which obviously does not give a full picture of the whole body antioxidant status.

To conclude, the study indicated the necessity of performing the MNA test for elderly upon admission to hospitals, and more attention needs to be paid to this vulnerable group of people. Detection of malnutrition in elderly subjects, upon admission to hospital, may assist in recovery and shortening hospital stay; consequently helping in reducing the hospital cost.[43,44]
